# The Relationship between Restrained Eating, Body Image, and Dietary Intake among University Students in China: A Cross-Sectional Study

**DOI:** 10.3390/nu13030990

**Published:** 2021-03-19

**Authors:** Cuiting Yong, Hanmei Liu, Qiping Yang, Jing Luo, Yufeng Ouyang, Minghui Sun, Yue Xi, Caihong Xiang, Qian Lin

**Affiliations:** Department of Nutrition Science and Food Hygiene, Xiangya School of Public Health, Central South University, 110 Xiangya Rd, Changsha 410078, China; yongcuiting@csu.edu.cn (C.Y.); hanmeiliu@csu.edu.cn (H.L.); yangqiping12@csu.edu.cn (Q.Y.); luojing2546@csu.edu.cn (J.L.); oyyf0102@csu.edu.cn (Y.O.); sun.1234@csu.edu.cn (M.S.); xiyue0404@csu.edu.cn (Y.X.); xch0622@csu.edu.cn (C.X.)

**Keywords:** restrained eating, body image, body dissatisfaction, dietary intake, dietary diversity scores

## Abstract

This study aims to explore the association between restrained eating, body image, and dietary intake among Chinese college students. This cross-sectional study included 1301 college students at two universities in Hunan Province. Electronic questionnaires were used to collect information including students’ demographic characteristics, restrained eating, perception of body weight, body satisfaction, and dietary intake. Anthropometric measurements were collected to calculate body mass index (BMI). The prevalence of high restrained eating was 52.8%. Students who were dissatisfied with their bodies or overestimated their body weight showed a higher tendency toward high restrained eating (*p* < 0.05). Students with high restrained eating tended to eat fruits and eggs more frequently, while the frequency of eating domestic animals and poultry, sugar-sweetened beverages, and fast food were the opposite. Moreover, high restrained eating was a risk factor for low dietary diversity (odds ratio (OR) = 1.384, 95% confidence interval: 1.002~1.912). The high incidence of restrained eating among Chinese college students and its energy-restricted diets that may lead to possible health implications require attention. Further studies are needed to investigate the characteristics of college students’ restrained eating to tailor appropriate interventions for forming positive body images and promoting healthy eating behaviors, thus, improving dietary quality.

## 1. Introduction

The concept of restrained eating was first presented by Herman in 1975 and defined as the tendency to restrict food intake to control weight [1]. The study of restrained eating has continuously progressed over time and led to a more comprehensive understanding. Taken together, it has several characteristics: (1) the purpose is to control weight, which includes two aspects—maintaining and losing weight; (2) the restriction behavior is characterized by cognitive and long-term features; (3) restrained eaters pay more attention to extrinsic food cues instead of intrinsic biological cues; and (4) it is more common in young women [2].

Studies show that restrained eating can predict the severity of eating disorders and is a key factor in their development [3,4]. On one hand, restrained eaters tend to reduce energy intake to maintain or lose weight [5], which makes them prefer low-calorie food, such as vegetables or fruits, and restrict high-calorie food intake, such as cereals and tubers or domestic animals and poultry [6]. Therefore, over-restrained eating may lead to inadequate energy intake. On the other hand, restrained eaters are more likely to overeat on some occasions, which is called the disinhibition effect, causing a range of eating disorder symptoms (e.g., bulimia nervosa, binge eating) [7]. Moreover, restrained eating is accompanied by recurrent and prolonged dieting and overeating, which brings negative emotions (e.g., depression, anxiety, and stress), severely impacting physical and psychosocial health [8,9].

College freshmen comprise an important group undergoing emerging adulthood in a critical period of life during which eating behavior and food preferences are changing and being gradually established, often persisting over time [10]. They have just begun a new journey, facing a richer dietary environment with more options of food. If not properly guided, new students are vulnerable to negative emotionality and external environmental changes; therefore, they are prone to developing poor eating habits and have an increased risk of eating disorders [11]. Some studies have indicated that college students have high restrained eating levels [11,12,13]. The study of restrained eating among Chinese college students is limited. By the end of 2019, there were more than 42 million enrolled college students in China. Hence, investigating restrained eating and dietary intake in Chinese college students is of eminent importance for the risk assessment of eating disorders.

Some studies have shown that body dissatisfaction or misperception of body weight is an important cause of restrained eating [14,15,16,17,18]. This may be related to the social norm that thinness is considered a mark of beauty, which may influence one’s attitudes and behaviors [19,20]. College students are in a critical period of aesthetic formation and are vulnerable to external influences. Studies have shown that the incidence of dissatisfaction with students’ body image and misperceptions of body weight are both at high levels [12,16]. However, only one study has discussed the moderating effect of body dissatisfaction on the relationship between BMI and dietary restriction among Chinese college students [14]. This research used self-reported weight and height, and did not discuss restrained eating by different body dissatisfaction levels. Therefore, the purpose of our study is to investigate the level of restrained eating among Chinese college students through the Three-Factor Eating Questionnaire, determine the incidence of misperception of body weight and body dissatisfaction, and analyze the relationship between restrained eating and body image.

## 2. Materials and Methods

### 2.1. Ethical Approval

The study was approved by the Ethics Committee of Xiangya School of Public Health, Central South University. The ethical batch number was XYGW-2019-2016, and it was pre-registered in the Chinese Clinical Trial Registry with the registration number ChiCTR-TRC-14005117. Before the survey began, all students in the study signed an informed consent form.

### 2.2. Study Design and Participants

This study is cross-sectional. From May to September 2019, the researchers recruited 1301 college students from two comprehensive universities in Changsha, Hunan Province, China. Inclusion criteria were as follows: (1) being undergraduates enrolled between September 2017 and September 2018; (2) having good reading comprehension ability; (3) being willing to accept the investigation and complete the questionnaire survey and physical examination. Exclusion criteria: having an acute or infectious disease at the time of investigation.

We used cluster sampling to randomly select three majors from three major categories of science and technology, liberal arts, and medicine in each university, and then selected one class from each freshman and sophomore in each major randomly, and took all students in the class as the research object. After obtaining informed consent, data were collected through electronic questionnaires. We used two methods of sample recruitment: (1) asking the tutors of each class to inform the students about the survey and (2) posting posters on campus to publicize the study.

### 2.3. Measures

#### 2.3.1. Questionnaire Survey

College students who agreed to participate in the survey scanned the QR code provided by the research group to read the electronic informed consent through their smart phones, and continued to visit and fill in the electronic questionnaire after choosing “agree.” In addition to demographic information, restrained eating, anxiety symptoms, depression symptoms, perceived stress, physical activity, perception of body weight, body satisfaction, and food consumption were also examined in this way.

- Demographic characteristics: Including the student’s gender, age, registered residence, ethnicity, sibling status (only child (yes vs. no)), academic major, and monthly living expenses, etc.
- Restrained eating: Restrained eating was investigated using the cognitive restraint dimension of the Chinese version of the Three-Factor Eating Questionnaire (TFEQ-18V3) [21], which was revised by TFEQ-R21 [22] among Chinese college students. The Cronbach’s alpha of TFEQ-18V3 was 0.87, and the retest reliability coefficient was 0.80. The Cronbach’s alpha and retest reliability coefficient of the cognitive restrained eating dimension were 0.76 and 0.79, respectively. The scale comprises 18 items rated with a four-point response scale. The cognitive restrained eating dimension includes five items, and the score ranges from 5 to 20. The total score of each dimension was finally converted into a 0–100 scale (final score = 100 × [original score − theoretical lowest score] ÷ theoretical score range). Higher scores indicate more intense CR (cognitive restrained eating). According to the research of Camilleri, G. M., CR scores were categorized based on the sex-specific median cut-points (excluding those with no CR): (1) no CR (score = 0); (2) low CR (0 < score < median); and (3) high CR (score ≥ median) [23].
- Perceived stress: Perceived stress scale-4 (PSS-4) was used to evaluate college students’ stress symptoms [24]. PSS-4 has been proven to have good reliability and validity in the Chinese population [25]. There are five items in the scale, and each item includes five categories of response (“never, hardly, occasionally, often, and very much,” rated 0 to 4, respectively). The score ≥ 90th percentile was defined as having stress symptoms [26]. A higher score means a higher degree of stress symptoms.
- Anxiety symptoms: Generalized Anxiety Disorder-2 (GAD-2) was used to investigate the anxiety status of college students [27]. GAD-2 consists of two items on a four-point response scale (from 0, never, to 3, almost every day). An overall score greater than 3 indicates the presence of anxiety [27]. GAD-2 has been proven to have good reliability and validity in the Chinese population [28,29].
- Depressive symptoms: Patient Health Questionnaire-9 (PHQ-9) was used to assess whether students had experienced depressive symptoms in the past two weeks [30]. The PHQ-9 consists of nine items on a four-point response scale with scores ranging from “0” to “3” respectively (“None”; “Few days”; “More than half of the days”; “Almost every day”). The overall score ranges from 0 to 27, with a higher score meaning a higher degree of depressive symptoms. Scores greater than or equal to 10 indicate the presence of depressive symptoms [30]. The Cronbach α reliability coefficient of the items was 0.89.
- Physical activity: Physical activity was assessed using the short-form self-administered instruments of the International Physical Activity Questionnaire (IPAQ) [31]. The questionnaire consisted of seven items and used indicators in MET min/week (Metabolic equivalence, MET) to indicate the intensity of physical activity. The IPAQ was scored according to recommended guidelines [32]. The physical activities were categorized into low, moderate, and high, based on IPAQ guidelines. Physical activity levels were divided into three grades: low activity or inactivity, moderate physical activity, and high physical activity [32].
- Satisfaction with body image: Our survey investigated Chinese college students and their satisfaction with their body image, and the results were divided into two categories: “satisfied or indifferent” and “dissatisfied.”
- Perception of body weight: According to the BMI standard of Chinese adults, the results were divided into four groups: underweight, normal, overweight, and obese. The participants’ perceptions of their weight were compared to their actual BMI category, and the results were divided into three groups: correct estimates, underestimates, and overestimates.
- Food consumption: The Food Frequency Questionnaire (FFQ) was used to investigate students’ food intake in the past 12 months. FFQ includes 32 kinds of food items, including nine categories of food, namely cereals and tubers, vegetables, fruits, domestic animals and poultry, aquatic products, beans and their products, dairy, eggs, and nuts. The response format ranged from 1, “Never eat,” to 9, “Eat three times a day.” Then, the intake frequency of 32 kinds of food was recoded into times per week: never eaten = 0, <1 time/month = 0, 1–3 times/month = 0.5 times/week, 1–2 times/week = 1.5 times/week, 3–4 times/week = 3.5 times/week, 5–6 times/week = 5.5 times/week; 1 times/day = 7 times/week, 2 times/day = 14 times/week, 3 times/day or more = 21 times/week or more. After that, food items were added according to food groups to calculate the average weekly consumption frequencies of each food group and to analyze the correlation between frequency of food intake and restrained eating. Moreover, we used dietary diversity scores (DDS) to evaluate students’ dietary quality. When calculating for DDS, points can be scored if at least one food in each category is consumed once a week or more. Consuming the same type of food scored for only one point so that the highest score was 9 points. Snacks, such as sugar-sweetened beverages, were not scored. Referring to Meng et al.’s study, >8 was evaluated as a good level of dietary diversification, while ≤8 was evaluated as low dietary diversity [33].

#### 2.3.2. Anthropometric Measurements

Height and weight of each participant were measured by the Tanita Body Composition Analyzer BC-W02C (No. 2210704, 2014), and Body mass index (BMI, kg/m^2^) was calculated based on these data. According to the BMI standard of Chinese adults, the healthy BMI range is 18.5~23.9 kg/m^2^. Those who were below the healthy range were underweight, those whose BMIs ranged from 24.0–27.9 kg/m^2^ were overweight, and those who were above 28.0 kg/m^2^ belonged to the obese group.

### 2.4. Statistical Analyses

We used SPSS 24.0 software (IBM Corp., Armonk, NY, USA) for statistical descriptions, chi-squared tests, Spearman rank correlation tests, and logistic regression analyses. Chi-squared tests were used to analyze students’ demographic characteristics and determine the relationship between restrained eating, body dissatisfaction, and perception of body weight. Spearman rank correlation tests were used to evaluate the correlation between restrained eating and weekly food consumption. Binary logistic regression was performed to explore the relationship between CR and dietary diversity, and 95% confidence intervals (CI) were calculated, while an odds ratio (OR) was estimated. The significance level was set at *p* < 0.05.

## 3. Results

### 3.1. General Characteristics of Participants

A total of 1307 college students participated in the survey, six unreliable information materials were eliminated, and 1301 valid data were obtained, with an effective response rate of 99.5%. Table 1 shows the basic information of college students. Among them, 508 (47.9%) were male and 793 (52.1%) were female, aged 17–25 (mean = 19.8, SD = 0.9). Of the students, 46.7% were registered in cities; 45.7% of the students were only-children. There was a significant difference in BMI between males and females (*p* < 0.001). The mean BMI was 21.4 kg/m^2^ (SD = 3.9), which was higher among males (22.5 ± 4.0 kg/m^2^) than females 20.8 ± 3.6 (20.8 ± 3.6 kg/m^2^, *p* < 0.001). Twenty-eight percent of college students overestimated their body weight, among which female students did so significantly more than male students (80.2% vs. 19.8%, *p* < 0.001), while 10.3% of them underestimated their body weight, among which male students did so significantly more than female students (73.0% vs. 23.0%, *p* < 0.001). Of the college students, 61.6% were not satisfied with their body image, among which females were significantly greater than males (64.8% vs. 35.2%, *p* < 0.001) (Table 1).

### 3.2. Variables of the Three-Factor Eating Questionnaire by Sex

We used the Three-Factor Eating Questionnaire to investigate the eating behavior of Chinese college students. The results showed that the mean score of cognitive restricted eating was 11.32 ± 3.20, and the score of female students was higher than that of male students (11.78 ± 3.13 vs. 10.58 ± 3.15, *p* < 0.001). The same is true for the score distribution of emotional eating and uncontrolled eating, with girls scoring higher than boys on average (Table 2).

### 3.3. Association between Cognitive Restraint, Emotion, Perception of Body Weight, and Satisfaction with Body Image

In this study, 687 college students had high restrained eating levels, accounting for 52.8% of the total. Students with a higher BMI tended to have higher levels of restrained eating. Students who overestimated their body weight were more likely to have a high level of restrained eating (60.8% vs. 39.2%, *p* < 0.001), and those who were not satisfied with their body image were more likely to have a high level of restrained eating (61.0% vs. 39.0%, *p* < 0.001). No association was observed between perceived stress, anxiety symptoms, depressive symptoms, and restrained eating (Table 3).

### 3.4. Correlation between Cognitive Restraint Eating and Weekly Consumption Frequency of Food and Snacks

Spearman rank correlation tests were used to analyze the correlation between restraint eating and weekly consumption frequency of food and snacks. Results showed that a high level of restrained eating was related to higher frequencies of eating eggs (*p* < 0.01) and fruits (*p* < 0.001) and lower frequencies of eating domestic animals and poultry, sugar-sweetened beverages, and fast food (*p* < 0.001) (Table 4).

### 3.5. Dietary Diversity Score by Cognitive Restraint

Binary logistic regression analysis was conducted with a “dietary diversity score ≤ 8” (reference = “> 8”) as the dependent variable to explore the relationship between restricted eating and dietary diversity. College students with high levels of restrained eating were more likely to have low levels of dietary diversity compared to those with no or low levels of restrained eating (OR = 1.384, 95% CI: 1.002~1.912) (Table 5, model 2). This association remained after adjusting for emotional and uncontrolled eating.

## 4. Discussion

The main purpose of this study was to investigate the relationship between restrained eating, body image, and dietary intake among Chinese college students through the Three-Factor Eating Questionnaire. The results showed that there were 687 college students with high restrained eating levels, accounting for 52.8% of the total. The mean restrained eating score was 11.32 ± 3.20, which was consistent with the results from other countries and regions [34,35]. Moreover, other studies using median or quartile to describe restrained eating have shown a high prevalence of high-level restrained eating in the population [36,37,38]. However, it is necessary to unify the two methods before comparing the results. Furthermore, our study found that female students scored significantly higher than male students (11.78 ± 3.13 vs. 10.58 ± 3.15, *p* < 0.001). Similar results have been found in studies of adults in France, Britain, and Finland [35,37,39]. This may be related to the more demanding social expectations of women’s body image [40]. It also could have something to do with the fact that women are more likely to be interested in energy-dense foods such as sweets, and, thus, more likely to engage in food-restricting behavior [41]. Our study also found that the scores of both emotional eating and uncontrolled eating among Chinese college students were higher for women than men, while the results of previous surveys were inconsistent [42,43].

In this study, college students with higher BMIs were more likely to be highly restrained eaters, and such a positive association was also found in other studies [44,45]. There are different explanations for these results. Being overweight is usually considered to be the result of restrained eating: the restraint theory suggests that dieters who try to cognitively control their food intake are prone to lose control of their diet and even turn to overeating when their cognitive process is disturbed (disinhibition phenomenon) [46]. These factors include depressive emotions and the intake of delicious food and alcohol [47,48]. Apart from disinhibition, it could also be explained in part by the fact that heavier people are more likely to diet [49]. Studies have shown that a higher BMI predicts dietary restraint, while dietary restraint does not predict future weight change. Restrained eating is, therefore, a marker of past weight gain, rather than a cause of future weight gain [50]. In a follow-up study of 3297 Finnish men and women aged 25 to 74 years, baseline restrained eating was not associated with a change in BMI at seven years. On the contrary, a higher baseline BMI predicted a larger increase in dietary restriction at seven years [51]. The possible explanation for restrained eating not predicting weight loss is that dieters were not dieting enough to reach the purpose of weight loss [50,52]. For example, they were not strict enough in reducing their food intake: although they ate less than they wanted, they actually ate no less than their physiological needs and not enough to have an effect on their weight [53].

When comparing self-estimated BMI with measured BMI, we found that college students who overestimated their body weight accounted for 28.0% of the total number of students, and those who were not satisfied with their body image accounted for 61.6% of the total number of students (Table 1). Moreover, students who overestimated their body weight (60.8% vs. 39.2%, *p* < 0.001) or were dissatisfied with their body image were both more likely to have high dietary-restraint tendencies (61.0% vs. 39.0%, *p* < 0.001) (Table 3). Meanwhile, students not satisfied with their body size were more likely to overestimate their body weight (see Appendix A, Table A1).

A study of Lebanese adults also found a link between body dissatisfaction and restrained eating and observed that pressure from social media to lose weight was also a contributing factor in restrained eating [18]. There may be a psychological explanation for this phenomenon. According to sociocultural theory, sociocultural factors play a central role in the development of human cognitive ability. Internalization is a core concept of sociocultural theory. This means that individuals demand their own behavior according to certain values and social standards formed in social culture [54]. The current social culture has shaped an ideal standard of thinness being beautiful, and this standard has become the social standard of individual image. Individuals internalize the ideal of slimness, and when there is a gap between their actual image and the ideal image, they will produce negative body images, which will lead to restrained eating behaviors [20,55]. 

According to social comparison theory, when there is no objective comparison standard, individuals will compare those people who are like them. Social comparison of body image has a significant impact on one’s self-evaluation [56]. Family, peers, and media factors are the most likely to lead to negative body image [57,58]. The pressure to be thin from family, peers, and the media together with the internalization of being thin as ideal creates conditions for a negative body image [59], which have been exacerbated by the development of new media such as We Media and social networking sites in recent years. Compared with traditional media, new media contain more socio-cultural pressures, which further exacerbates the cult of thinness [60]. 

Social and mass media used for interpersonal communication, including photos, video logs, and other personal information, are highly interactive. On social networking sites, people can make public comments on other peoples’ posts [61]. Moreover, publishing self-images have made women more likely to be judged by their peers over their appearance [62]. All these make social networking sites an important place for individuals to make social comparisons (mainly body image comparisons) [63]. The results show that girls who regularly share their self-images on social media tend to overestimate their body shape and weight and have higher levels of body dissatisfaction and dietary restriction compared to girls who do not use social media [64]. Social media use is also more strongly associated with body dissatisfaction than any other Internet-related activity [65].

Our study shows that in both college students who overestimate their body weight (80.2% vs. 19.8%, *p* < 0.001) and those who are dissatisfied with their body image (64.8% vs. 35.2%, *p* < 0.001), female students do so significantly more than male students. However, male students underestimated their body weight significantly more than did female students (73.0% vs. 23.0%, *p* < 0.001) (Table 1), with the social culture related to the different expectations for both men and women. 

These results have to do with the different cultural expectations of men and women. Women must be thin to be physically attractive. This social culture of thinness encourages women to try to become thinner, such as by restricting their diet. While the social culture generally requires men to be stronger, men who want to lose weight tend to use exercise as a means of weight control [40]. This is supported by research from other regions: a survey of the body size of college students in 22 different countries and regions found that more women than men identified themselves as overweight in any decile. Women tended to overestimate their weight, while men did not. Women were more likely to lose weight when they self-evaluated as overweight, while even men in the highest BMI decile were less aware of their overweight status and less likely to try to lose weight. The study also found that both men and women in Asian countries showed higher levels of dieting, which is related to the social culture of Asian countries, where people’s ideal weight is generally lower than in the United States and Europe [66].

We also investigated the relationship between restrained eating and dietary intake in college students. For sugar-sweetened beverages and fast food, the higher the restrained eating score, the lower the frequency of consumption (Table 4). The same results were found in a French study of 887 people [67], where high-level restrained eaters had lower intakes of sweets and sugar-sweetened beverages, and restrained eating was associated with reduced total energy intake [67,68]. This may be related to the fact that restrained eaters pay more attention to the calorie content of their food, which increases their motivation to suppress appetite and their ability to reduce unhealthy food intake [69]. 

Second, the subjects of our study were freshmen and sophomores, and the correlation between high restrained eating and decreased food cravings was more obvious in the younger group [34,70]. The study showed a much stronger negative association between the level of restrained eating and cravings for high-fat, sweet, and fast-food foods in the ≤25 age group than in the >25 age group, the sweets craving was strongly influenced by gender in the ≤25 age group, and the correlation was more pronounced in women [34,70].

In addition to the reduction in the intake of unhealthy foods, restrained eating was also associated with changes in the intake of healthy foods. We observed a negative correlation between the frequency of domestic animals and poultry intake and restrained eating scores, but the opposite was observed for fruits and eggs (Table 4). Another study of 596 female college students also found that the likelihood of participants following a lacto-ovo or vegan diet increased significantly as the level of restrained eating increased [38]. This may be related to the low energy and fat content of these foods, and restrained eaters are more inclined to choose these foods to control calorie and fat intake [5]. However, when comparing the levels of restrained eating between vegetarians and omnivores, the vegetarians had lower levels of restrained eating. A possible explanation is that in omnivores, restriction counteracts the effects of overeating and, thus, helps to control weight, whereas vegetarians, who have little inclination to overeat, have lower levels of restriction [71,72].

We also used the DDS for the first time to evaluate the dietary quality of restrained eating college students. The results showed that college students with high restrained eating were more likely to have low levels of dietary diversity than those with no or low restrictive eating (OR = 1.384, 95% CI: 1.002~1.912) (Table 5, model 2). Previous studies have shown that individuals with high restrained eating are more likely to choose low-energy and healthier foods, which tends to make them reduce energy-rich foods such as cereals and tubers, and domestic animals and poultry, and switch to vegetables and fruits, leading to a decrease in food diversity [70,73]. Meanwhile, excessive restriction of energy intake may also bring further negative health effects, such as the lack of carbohydrates and fat in vegetarian diets, which may lead to an increased incidence of menstrual disorders in women [38]. This association between high levels of dietary restraint and irregular menstruation may have further implications for women’s health. In addition to the obvious effects on fertility, bone health may also be affected; menstrual dysfunction can affect circulating steroids and is known to cause bone loss [74]. Moreover, a review of the health effects of low-carbohydrate diets showed that while short-term carbohydrate restriction of more than a week can lead to significant weight loss, this type of diet may pose potential health hazards over a longer period of months to years. Complications including arrhythmia, cardiac systolic dysfunction, sudden death, osteoporosis, kidney damage, increased risk of cancer, physical activity disorders, and lipid abnormalities may be related to long-term dietary carbohydrate restrictions [75].

Relevant studies have been carried out to intervene in restrained eating or dieting and the accompanying negative body image. Cognitive intervention based on school or community showed urging participants to correctly deal with sociocultural pressure and reducing their ideal internalization of thinness to be an effective intervention. It helps to promote participants to establish the correct perception of body weight and positive body image, form good eating habits and reduce the risk of eating disorders and related diseases [76,77]. However, the follow-up results show that the intervention effect is not clear. Long-term and more intensive interventions are still needed to lastingly change the body image and dieting behavior [76,77]. Another 8-week intervention in college-age women with high weight and body size concerns showed that Internet-based cognitive behavioral intervention could significantly reduce weight and body size concerns for up to two years and reduce the incidence of eating disorders [78]. Other effective interventions include nutrition education and theory-based behavior change interventions [79]. It is worth noting that in a physical activity intervention designed to prevent dieting and excessive concerns about weight, physical activity interventions may increase concerns about women’s slimness; therefore, health promotion research should include the assessment of potential negative side effects [80].

This study investigated the incidence of high-level restrained eating, body dissatisfaction, and misperception of body weight among freshmen and sophomores in China. The results help understand young people’s attitudes towards body image and provide a reference for guiding them to recognize body shape and establishing healthy eating behaviors correctly. We also used DDS for the first time to evaluate college students’ dietary quality and explored the relationship between restrained eating and dietary intake. The results provide a basis for the dietary guidance and nutrition education for college students with restrained eating and the formulation of dietary health-related policies in the future. 

However, the study has its limitations. First of all, due to the large sample size and content of our questionnaire and the limited survey time (between two categories), this study used FFQ as a diet survey tool. Considering the low accuracy of food intake self-estimates, we did not set items to investigate the amount of food consumed. Therefore, participants’ diet quality can only be roughly assessed by the frequency of food intake and DDS. Further research is needed to explore the impact of restrictive eating on the quality of students’ diets. Secondly, in the process of sample collection, due to the different degree of completion and participation rate of students, participants majoring in science and technology account for a high proportion of the sample population. Therefore, this sample cannot reflect the restrained eating behaviors, body image, and dietary intake of all Chinese college students. Finally, only cross-sectional data were collected in this study, so it is not possible to determine the sequence and possible interaction between restrained eating, body image, and dietary intake in students. In future studies, a large, prospective, population-based study should be conducted to follow up restrained eating behaviors in the sample and evaluate their interaction with body image and dietary intake.

## 5. Conclusions

In this study, we found that the incidence of high restrained eating among Chinese college students was at a high level. College students who overestimated their body weight or who were dissatisfied with their body image had a higher tendency to restrain their eating. Moreover, college students with high restrained eating may have controlled their dietary energy intake by reducing their intake frequency of energy-dense foods such as sugar-sweetened beverages and fast food and increasing their intake of low-energy foods such as eggs and fruits. Long-term adoption of such a calorie-restricted diet has been shown to have a variety of potential short- and long-term health implications; thus, the high prevalence of restrained eating among Chinese college students needs attention. Further studies are needed to investigate the characteristics of Chinese college students’ restrained eating to help tailor interventions targeting establishing correct perception of body weight and positive body image, forming healthy eating behaviors.

## Figures and Tables

**Table 1:** General characteristics of the participants.

Variables	N	Male (n%)	Female (n%)	χ^2^		*p*
Total	1301	508 (39.0%)	793 (61.0%)			
Age				29.858		0.000
≤18 years	195 (15.2%)	53 (27.2%)	142 (72.8%)			
19 years	598 (46.6%)	213 (35.6%)	385 (64.4%)			
20 years	373 (29.0%)	173 (46.4%)	200 (53.6%)			
≥21 years	118 (9.2%)	60 (50.8%)	58 (49.2%)			
Registered residence				0.209		0.647
Urban	694 (53.3%)	275 (39.6%)	419 (60.4%)			
Rural	607 (46.7%)	233 (38.4%)	374 (61.6%)			
Only child				12.241		0.000
Yes	595 (45.7%)	263 (44.2%)	332 (55.8%)			
No	706 (54.3%)	245 (34.7%)	461 (65.3%)			
Major				68.661		0.000
Liberal arts	179 (13.8%)	27 (15.1%)	152 (84.9%)			
Science and technology	840 (64.6%)	390 (46.4%)	450 (53.6%)			
Medicine	160 (12.3%)	48 (30.0%)	112 (70.0%)			
Others	122 (9.4%)	43 (35.2%)	79 (64.8%)			
Monthly expenditure				13.323		0.004
≤500 RMB	20 (1.6%)	15 (75.0%)	5 (25.0%)			
500–1000 RMB	265 (20.7%)	109 (41.1%)	156 (58.9%)			
1000–2000 RMB	897 (69.9%)	343 (38.2%)	554 (61.8%)			
>2000 RMB	101 (7.9%)	33 (32.7%)	68 (67.3%)			
BMI				64.888		0.000
Underweight	158 (16.1%)	41 (25.9%)	117 (74.1%)			
Normal	673 (68.7%)	238 (35.4%)	435 (64.6%)			
Overweight	114 (11.6%)	81 (71.1%)	33 (28.9%)			
Obesity	34 (3.5%)	15 (44.1%)	19 (55.9%)			
Physical activity				13.266		0.001
Low	276 (21.2%)	104 (37.7%)	172 (62.3%)			
Median	793 (61.0%)	289 (36.4%)	504 (63.6%)			
High	232 (17.8%)	115 (49.6%)	117 (50.4%)			
Perceived stress				1.232		0.267
Yes	73 (5.6%)	33 (45.2%)	40 (54.8%)			
No	1228 (94.4%)	475 (38.7%)	753 (61.3%)			
Anxiety symptoms				0.286		0.593
Yes	159 (12.2%)	59 (37.1%)	100 (62.9%)			
No	1142 (87.8%)	449 (39.3%)	693 (60.7%)			
Depressive symptoms				3.336		0.068
Yes	242 (18.6%)	107 (44.2%)	135 (55.8%)			
No	1059 (81.4%)	401 (30.8%)	658 (50.6%)			
Perception of body weight				92.096	0.000	
Underestimate	100 (10.3%)	73 (73.0%)	27 (27.0%)			
Correct	601 (61.7%)	244 (40.6%)	357 (59.4%)			
Overestimate	273 (28.0%)	54 (19.8%)	219 (80.2%)			
Satisfaction with body image				13.259	0.000	
Satisfied	499 (38.4%)	226 (45.3%)	273 (54.7%)			
Not satisfied	802 (61.6%)	282 (35.2%)	520 (64.8%)			

Chi-square test was performed to compare the groups. BMI: body mass index; RMB: Renminbi, Chinese official coupons, 1 RMB ≈ 0.16 USD.

**Table 2:** Variables of The Three-Factor Eating Questionnaire by sex (x ± SD).

Variables	Total	Male	Female	*t*	*p*
Cognitive restraint	11.32 ± 3.20	10.58 ± 3.15	11.78 ± 3.13	−6.737	0.000
Emotional eating	12.64 ± 4.51	11.17 ± 4.35	13.58 ± 4.36	−9.746	0.000
Uncontrolled eating	15.75 ± 4.39	14.57 ± 4.60	16.51 ± 4.08	−7.737	0.000

*t* test was performed to compare the groups. SD: Standard deviation.

**Table 3:** Association between CR, emotion, perception of body weight and satisfaction with body image.

Variables	N	No CR/Low CR (n%)	High CR (n%)	χ^2^	*p*
Total	1301	614 (47.2%)	687 (52.8%)		
Perceived stress				0.378	0.539
Yes	73 (5.6%)	37 (50.7%)	36 (49.3%)		
No	1228 (94.4%)	577 (47.0%)	651 (53.0%)		
Anxiety symptoms				0.451	0.502
Yes	159 (12.2%)	79 (49.7%)	80 (50.3%)		
No	1142 (87.8%)	535 (46.8%)	607 (53.2%)		
Depressive symptoms				0.293	0.589
Yes	242 (18.6%)	118 (48.8%)	124 (51.2%)		
No	1059 (81.4%)	496 (46.8%)	563 (53.2%)		
Perception of body weight				18.620	0.000
Underestimate	100 (10.3%)	63 (63.0%)	37 (37.0%)		
Correct	601 (61.7%)	302 (50.2%)	299 (49.8%)		
Overestimate	273 (28.0%)	107 (39.2%)	166 (60.8%)		
Satisfaction with body image				55.964	0.000
Satisfied	499 (38.4%)	301 (60.3%)	198 (39.7%)		
Not satisfied	802 (61.6%)	313 (39.0%)	489 (61.0%)		
BMI				53.883	0.000
Underweight	158 (16.1%)	116 (73.4%)	42 (26.6%)		
Normal	673 (68.7%)	306 (45.5%)	367 (54.5%)		
Overweight	114 (11.6%)	41 (36.0%)	73 (64.0%)		
Obesity	34 (3.5%)	10 (29.4%)	24 (70.6%)		

CR: cognitive restrained eating; BMI: body mass index; Chi-squared test was performed to compare the groups.

**Table 4:** Correlation between cognitive restraint eating and weekly consumption frequency of food and snacks.

Food Group	Median (P25, P75)	Correlation Coefficient	*p*
Cereals and tubers	22.0 (16.0, 29.0)	−0.041	0.140
Vegetables	12.0 (6.0, 19.5)	0.030	0.286
Fruits	9.5 (4.5, 17.0)	0.100	0.000
Domestic animals and Poultry	10.5 (6.0, 15.5)	−0.129	0.000
Aquatic products	1.0 (0.0, 2.0)	−0.020	0.462
Beans and their products	5.0 (2.0, 7.0)	−0.001	0.966
Dairy	3.5 (1.5, 5.5)	0.034	0.223
Eggs	3.5 (1.5, 5.5)	0.075	0.006
Nuts	0.5 (0.0, 1.5)	0.029	0.297
Fast food	1.5 (0.5, 3.0)	−0.146	0.000
Sugar-sweetened beverages	1.5 (0.5, 3.5)	−0.249	0.000

Spearman rank correlation test was performed to analyze the between restraint eating and food consumption frequencies.

**Table 5:** Logistic regression analysis of the related factors of dietary diversity scores (DDS) among participants.

Variables	Dietary Diversity Score ≤ 8 OR (95 %CI)		
	Crude OR	Model 1	Model 2
Cognitive restraint (reference = “No CR/Low CR”)			
High CR	1.395 (1.038, 1.875) *	1.430 (1.042, 1.962) *	1.384 (1.002, 1.912) *
Emotional eating (reference = “No EE/Low EE”)			
High EE	1.313 (0.978, 1.763)		1.157 (0.828, 1.617)
Uncontrolled eating (reference = “No UE/Low UE”)			
High UE	1.232 (0.915, 1.658)		1.135 (0.812, 1.585)

Model 1: adjusted for sex, age, registered residence, monthly expenditure, major, physical activity, perceived stress, anxiety symptoms, depressive symptoms, and satisfaction to body image; Model 2: additionally adjusted for emotional eating and uncontrolled eating; * *p* < 0.05; EE: emotional eating; UE: uncontrolled eating. OR, odds ratio. 95% CI, 95% confidence interval.

## Data Availability

The data that support the findings of this study are not publicly available due to the data containing information that could compromise participant privacy but are available from the corresponding author on reasonable request.

## Appendix A

**Table A1:** Perception of body weight and BMI by satisfaction with body image.

Variables	N	Satisfied with Body Image (n%)	Not Satisfied with Body Image (n%)	χ^2^	*p*
Total	1301	370 (38.0%)	604 (62.0%)		
Perception of body weight				34.999	0.000
Underestimate	100 (10.3%)	48 (48.0%)	52 (52.0%)		
Correct	601 (61.7%)	258 (42.9%)	343 (57.1%)		
Overestimate	273 (28.0%)	64 (23.4%)	209 (76.6%)		
BMI				43.784	0.000
Underweight	158 (16.1%)	89 (56.3%)	69 (43.7%)		
Normal	673 (68.7%)	255 (37.9%)	418 (62.1%)		
Overweight	114 (11.6%)	22 (19.3%)	92 (80.7%)		
Obesity	34 (3.5%)	7 (20.6%)	27 (79.4%)		

Chi-square test was performed to compare the groups; BMI: body mass index.
